# Essure® present controversies and 5 years’ learned lessons: a retrospective study with short- and long-term follow-up

**DOI:** 10.1186/s10397-017-1023-3

**Published:** 2017-10-03

**Authors:** Sara Câmara, Filipa de Castro Coelho, Cláudia Freitas, Lilia Remesso

**Affiliations:** Department of Obstetrics and Gynecology, Hospital Dr. Nélio Mendonça, Avenida Luís de Camões nº 57, Funchal, 9004-514 Portugal

**Keywords:** Hysteroscopy, Sterilization, Counseling, Patient satisfaction, Pelvic pain

## Abstract

**Background:**

The risk-benefit of contraception with Essure® is being readdressed due to an increase of reports of adverse effects with this device. Our aim was to proceed to an internal quality evaluation and to identify opportunities for protocol improvement.

We proceeded to a one-center, retrospective consecutive case series of women admitted for Essure® placement, from 1 January 2012 until 31 December 2016 (5 years).

**Results:**

In a total of 274 women, technical difficulties were mainly unilateral, with no acute or short-term severe complications. The procedure was brief (median 3.2 min, IQR 2.5–5.2) and moderately painful (median of 4 in a 0–10 scale; IQR 3–5). At 3 months, the failure rate was 2%, with no pregnancies. Second surgery indication (< 1%) resumed to a case of nickel hypersensitivity. At 1 year, pregnancy rate was 1%. Ninety-eight percent of the patients would recommend the method.

**Conclusions:**

We identified high patient satisfaction and low failure rates, both at short and long term. Investigation about whether some women still have patent tubes at the 3-month follow-up could lead to protocol improvement. It is important that clinicians look for second causes for adverse effects related to Essure® and avoid the erroneous indication for implant removal. Long follow-up allowed for both internal quality evaluation and clarification of misconception; it could possibly also have contributed to patient satisfaction.

## Background

Office hysteroscopic sterilization is more cost-effective than laparoscopic sterilization and is a better option in high-operative-risk women [1]. Its success depends on careful patient selection, surgeon’s experience, reliable compliance of back-up contraception, and observance of the 3-month follow-up [2]. Recently, there has been an imperative urge to survey Essure® risk-benefit profile during its life cycle, due to an intensification of adverse effect report [2, 3].

While being a highly satisfactory permanent contraception method, pelvic pain (related or not to menses) and irregular bleeding or heavy menstruation are among the most frequent late complications related to the procedure [2]. However, these symptoms are not always related to the micro-inserts itself but to other underlying gynecologic conditions (endometriosis, adenomyosis, or others), which could erroneously indicate the implant surgical removal [2, 4].

Several studies have been reported which describe long-term follow-ups (from 1 to 5 years) of patients submitted to sterilization with Essure® [5–7]. In this study, the authors have proceeded to a retrospective consecutive case series analysis of *one-center* (*two hysteroscopists*), 5 years’ experience with Essure®. The authors’ aim was first to evaluate internal quality (procedure-associated surgical difficulties; patient acute complications; failure and pregnancy rates; adverse effects; and satisfaction at long follow-up) and second, to identify opportunities for protocol improvement.

## Methods

A retrospective case series analysis of women admitted to hysteroscopy with intention to treat for Essure® placement, at our center, from 1 January 2012 until 31 December 2016, was done.

Patient selection always included a previous standardized initial appreciation and information on this permanent contraception method (Table 1). All women received contraceptive counseling and were clinically evaluated by one of the two hysteroscopists, who would realize the procedure. Premedication with paracetamol or with non-steroidal anti-inflammatory was used at patient discretion, and this information could not be retrospectively confirmed. At all times, the placement of the micro-inserts was accomplished by vaginoscopy, without anesthesia or cervical dilatation. Women who were not using contraception at this moment or who were using barrier methods would be advised to start a hormonal contraceptive pill for back-up contraception, except if contraindicated.

**Table 1:** Previous counseling included items

▪ Full gynecological evaluation (clinical history, gynecological observation, transvaginal ultrasound, and cervical and breast cancer screening if indicated)	
▪ Confirming the motivation to permanent contraception	
▪ Evaluation of nickel or metal allergy	
▪ Full information on the procedure (anatomical and technical details)	
▪ Full information on possible complications and restrictions	*Early*: infection, acute pain, perforation/migration/expulsion
	*Late*: chronic pain or irregular/heavy bleeding
	*Restrictions*: ✓ Magnetic resonance—safe if using a 1.5 T magnet; artifact possibility ✓ Electrosurgical procedures—should be avoided if near the micro-inserts

Collected variables were:

- demographic characteristics (age, gravidity, parity, contraceptive method in use);
- procedure description (surgeon’s perceived difficulty; duration of the procedure from the hysteroscope entrance until its exit from the uterine cervix external ostium; patient reported pain immediately after the procedure, in a numeric scale of 1–10);
- follow-up at 3 months (consisting of an appointment with the hysteroscopist of reference to monitor early complications; confirm correct localization of the micro-inserts either by X-ray, gynecological ultrasound, hysterosalpingography, or hysterosonography; and inform the patient if the back-up contraception should be prolonged or abandoned);
- and follow-up after the first completed year (women were contacted by phone by the same medically trained operator and were asked to classify their satisfaction with the method as “very unsatisfied,” “unsatisfied,” “satisfied,” or “completely satisfied” to state reasons in case they were not completely satisfied and if they would likely recommend this method).

Finally, failure (defined by incorrect micro-implant localization or tubal permeability) rates and pregnancy rates at 3 months and 1 year were calculated, and second surgery requirement and indication were explored.

## Results

### Essure® delivery

During 5 years, 274 women were admitted to hysteroscopy with intention to treat for Essure® delivery (Table 2).

**Table 2:** Demographic characteristics of patients submitted to hysteroscopy for Essure® delivery

Age (years)	mean = 38 (SD = 4; range 27–46)
Gravidity	median = mode = 2 (IQR 2–3; range 0–7)
Parity	median = mode = 2 (IQR 2–3; range 0–6)
Previous contraceptive method	▪ SARC users: 65% (*n* = 178), of which 96% (*n* = 171) were using a contraceptive pill ▪ LARC users 15% (*n* = 42) ▪ Others (barrier or natural method of contraception) 20% (*n* = 54)

*SARC* short-acting reversible contraception, *LARC* long-acting reversible contraception

This was not achieved bilaterally in 7.3% of the patients mainly because of obstruction to the delivery catheter progression but also because of non-identified ostium or ostia (Table 3)*.* The minority of patients with non-identified ostium or ostia were using continuous hormonal contraception (either a combined estroprogestative or a progestative-only pill). Device (manufacturer)-related difficulties were not identified. Women who could not be successfully sterilized with Essure® underwent either laparoscopic tubal ligation or salpingectomy (according to the woman’s informed choice). There were no severe acute complications. The only acute complication was one case of vagal reaction, immediately after successful implant delivery (with complete satisfaction at long follow-up).

**Table 3:** Procedure description

Surgical difficulties	▪ Surgeon’s perceived difficulty in inserts delivery 11% (*n* = 31), of which 65% (*n* = 20) unilateral ▪ Successful placement not achieved in 7.3% (*n* = 20): ✓ tube obstruction because of spasm/stenosis (*n* = 16) ✓ non-identified ostium/ostia (*n* = 4)
Hysteroscopy duration (minutes)	∆*t* median 3.2 (IQR 2.5–5.2; range 1–15)
Patient reported pain (0–10 scale)	median score = 4 (IQR 3–5; range 0–8); mode = 3

### Early (3 months) follow-up (*n* = 254)

For the 3-month follow-up, we considered the 254 women with successful delivered micro-inserts. No loss of follow-up or pregnancy was registered. In 2% (*n* = 4), the confirmatory exams revealed that the procedure was ineffective (two cases of contrast leakage; one case of leakage in the post-salpingectomy side, with confirmed contralateral obstruction; and one case of insert unilateral expulsion, brought in hand by the patient and confirmed by imaging). This last patient underwent subsequent placement of a new micro-insert which she again noticed to deliver per the vagina some days afterwards (with correct number of coils trailing in uterine cavity, namely three, bilaterally and without perceived surgical difficulty during its placement).

During this first trimester, there were no cases of fever, documented infection, or pelvic pain requiring further care.

One allergic reaction to nickel manifested shortly after the 3-month follow-up with a generalized dyshidrotic eczema, and this was confirmed by immunoallergology patch test. This woman had no history of allergy to metals, and the situation resolved completely after hysterectomy (which was the decision taken together with patient, considering the surgical risks of a more conservator approach, including that of incomplete device removal).

### Late (≥ 1 year) follow-up, complications, and satisfaction (*n* = 249)

Considering the patients with a 3-month follow-up which confirmed correct Essure® placement, a total of 249 patients had the implants for more than 1 year before. Not all these patients could be contacted by telephone at several attempts. At the end, 72% of them (*n* = 179) answered to the satisfaction survey; 88% (*n* = 158) were completely satisfied and 9% (*n* = 17) were very satisfied but reported abnormal menstrual bleeding and/or pelvic pain (related or not to menstruation). Two percent (*n* = 4) were unsatisfied or very unsatisfied and were the only who would not recommend this method of contraception (two of these women had the same symptoms than the other patients but more severe, requiring the reintroduction of hormonal therapy; the other two women had correct micro-insert placement documented by either X-ray and ultrasound or by hysterosalpingography but reported a pregnancy diagnosed at their private medical assistance). Assuming these two cases of pregnancy, the pregnancy rate at 1 year was 1%.

In this satisfaction survey, two women pointed out that they had been denied magnetic resonance. This question was readdressed and clarified during the satisfaction survey. Some women revealed misconceptions about the procedure (relating it to the posterior appearance of an adnexal mass or to abnormal cervical smear), which was elucidated at that moment.

## Discussion

Although there are presently many controversies about Essure®, evidence about long-term follow-up of patients submitted to this type of definitive contraception is scarce. The importance to proceed to an extended survey of patients’ satisfaction has been unanimously strengthened by previous studies [5–9]. In what concerns the demographic findings in our population, they are similar to what has been described by others [5–9].

### Surgical difficulty and unsuccessful procedure

In our experience, the surgeon perceived the procedure as difficult in 11% of the cases (mainly unilateral difficulty), with an unsuccessful micro-insert delivery (either with or without attempt), comparable to the results found in the literature (7.3 versus 1.5% [9], 2.8% [7], 8% [6], 12% [10]). Systematic use of a non-steroidal anti-inflammatory or a spasmolytic before the procedure (except if contraindicated) could possibly contribute to diminish tubal spasm and subsequent obstruction, but this is yet to be probed. Although we had no event of perforation or abdominal migration, these events could be related to tubal cannulation attempts and, therefore, also benefit from tubal spasm prevention. In what concerns continuous hormonal contraception, it is thought to improve visibility and therefore facilitate the implant delivery. Theoretically, continuous progestins, instead of combined hormonal contraception, may be more favorable due to a higher atrophic effect of the endometrium, but this is not evidence-based. In our sample, however, all women with non-identified ostium/ostia were using continuous hormonal contraception.

### Patient acute complications

Besides acute pain and one self-limited vagal reaction, we had no other incidents like perforation, syncope, or infection. In the literature, there is no evidence about acute complication incidence, but our findings and of others support that this procedure is moderately painful even if brief [5–8]. An incidence of 4.2% of pain chronification (lasting more than 3 months after the procedure) has been estimated [10]. Because our study is retrospective, we could not identify our incidence of chronic pain.

### Failure and pregnancy rates

A good adherence to the 3-month confirmatory tests and back-up birth control after hysteroscopic sterilization is important to avoid unintended pregnancies. Remarkably, we had no loss to follow-up or pregnancy events at 3 months, with a correct placement confirmed in 99% of the tests which is analogous to previous publications (99.5% [7] and 99.6% [9]; even if in these series a lower first control compliance rate is reported 90.8 and 96.8%, respectively). Our 3-month follow-up detected failures were due to contrast leakage or micro-insert expulsion. In the second case, we would now opt for another type of sterilization instead of a second attempt. In view of the fact that the implant expulsed had been delivered with no difficulty, that a correct number of coils trailed in the cavity, and the facility at which it was asymptomatically expulsed, we can imagine that there can be a sort of tubal incompetency.

At 1-year follow-up, we had two pregnancies reported (corresponding to a failure rate of 1%), after documented correct micro-inserts localization. Both, incorrect interpretation of those tests or correct interpretation of localization tests but without obstruction confirmation, are plausible explanations. However, it may also be hypothesized that some women may produce less or slower reaction to the implant, accounting for cases of migration or expulsion, or even for cases of pregnancy after the 3-month correct follow-up. In agreement with this hypothesis, higher than expected tubal patency at intervals less than 1 year [11] and cases of pregnancy after the 3-month ideal follow-up have been published [2, 7]. Failure rates of all types of sterilization failures are estimated in 0.9% [12] but there is uncertainty about specific failure rates for Essure® due to either short follow-up studies, discrepancy between study results, or lack of information on follow-up or on accomplished protocol [13, 14].

### Adverse effects and satisfaction at long follow-up

Complication rate for laparoscopic sterilization has been estimated in 1.6% [15]. In our study, we had no case of infection, perforation, embolus, or death. Considering the case of nickel hypersensitivity, our post-procedure second surgery was 0.4%. We consider that the suspicion of nickel allergy should contraindicate the procedure even if since 2011 this is no longer contraindicated by the Food and Drug Administration and if some authors would recommend further evaluation but nevertheless consider the procedure [7]. In the manufacturer’s official site, it can be read that *no* test can reliably predict this adverse effect [16].

Like in our study, studies evaluating women satisfaction have constantly found high levels of early and late satisfaction with levels of moderate or lower pain [6–8]. The most frequent reported secondary adverse effects are pelvic pain (with or without dysmenorrhea) and increased menses bleeding or irregularity [2]. Unfortunately, we could not assess *“*de novo” pain cases because most of these women were previously under hormonal contraception, which could have masked previous symptoms. Resuming hormonal therapy ameliorated the symptoms, avoiding the need for second surgery for implant removal (guaranteeing a more effective contraception). Others have enlightened the importance to clearly evaluate the complaints that patients relate to Essure®, at the risk of removing the implants without improvement [2, 4]. With reported second surgery rates of 0.4% [7] to 4.3% [4], according to a recent retrospective cohort study, more than half of surgeries post-Essure® placement were related to pain complaints and the majority of them related to subjacent gynecological conditions [4]. We believe that this finding is of great relevance for clinicians who evaluate women with Essure®.

### Final considerations

The most important weakness of our study is its retrospective nature. With our findings, we now look forward to proceed in our investigation in a prospective way and to a longer interval of follow-up.

Finally, we believe that the possibility to inform women about misconceptions of long-term effects of the method contributes to a higher patient satisfaction, though this has not been previously studied. Further investigation is needed to identify which benefits can be obtained by premedication and if a different first follow-up control could have higher sensitivity in the detection of the method failure.

## Conclusions

From 5 years of experience with Essure®, we identified very high rates of satisfaction with this extremely effective and accessible method, associated to moderate pain in a fast procedure, with a low need for second surgery. The unsatisfaction related to the method is usually due to misconceptions about it, which is why we attribute great relevance to prior candidate selection and specific counseling. Long follow-up allows for both further identification of method failure and for a second chance for misconception clarification. It can probably also contribute to a higher patient satisfaction.

## Abbreviations

LARC: Long-acting reversible contraception
SARC: Short-acting reversible contraception
